# Deep cultural ancestry and human development indicators across nation states

**DOI:** 10.1098/rsos.171411

**Published:** 2018-04-11

**Authors:** Roland B. Sookias, Samuel Passmore, Quentin D. Atkinson

**Affiliations:** 1 Museum für Naturkunde, Leibniz Institute for Evolution and Biodiversity Science, Invalidenstraße 43, 10115 Berlin, Germany; 2 School of Psychology, University of Auckland, Auckland, Private Bag 92019, Auckland 1142, New Zealand; 3 Department of Anthropology and Archaeology, University of Bristol, 43 Woodland Road, Bristol BS8 1UU, UK; 4 Max Planck Institute for the Science of Human History, Jena, Germany

**Keywords:** phylogenetics, cultural evolution, international development, Indo-European

## Abstract

How historical connections, events and cultural proximity can influence human development is being increasingly recognized. One aspect of history that has only recently begun to be examined is deep cultural ancestry, i.e. the vertical relationships of descent between cultures, which can be represented by a phylogenetic tree of descent. Here, we test whether deep cultural ancestry predicts the United Nations Human Development Index (HDI) for 44 Eurasian countries, using language ancestry as a proxy for cultural relatedness and controlling for three additional factors—geographical proximity, religion and former communism. While cultural ancestry alone predicts HDI and its subcomponents (income, health and education indices), when geographical proximity is included only income and health indices remain significant and the effect is small. When communism and religion variables are included, cultural ancestry is no longer a significant predictor; communism significantly negatively predicts HDI, income and health indices, and Muslim percentage of the population significantly negatively predicts education index, although the latter result may not be robust. These findings indicate that geographical proximity and recent cultural history—especially communism—are more important than deep cultural factors in current human development and suggest the efficacy of modern policy initiatives is not tightly constrained by cultural ancestry.

## 1. Introduction

### 1.1. Human development

Despite decades of global economic growth, there remain stark disparities in levels of development between nations, with significant implications for human welfare. The widely cited United Nations (UN) Human Development Index (HDI) [1] combines economic, health and education metrics to provide a proxy for human welfare designed to capture the extent to which residents of a country can enjoy long, healthy and meaningful lives. At one end of the spectrum, for example, Norway enjoys high *per capita* income (US$47 500) and life expectancy (81 years) and many attend school into early adulthood [2]. By contrast, in Bangladesh *per capita* income and life expectancy are much lower (US$1530 and 68 years) and people have on average less than five years of schooling [2]. While such measures cannot capture all facets of human welfare [3], identifying what factors predict metrics like HDI is important for understanding the forces shaping global development [4,5] and for guiding policy decisions [6,7].

### 1.2. Factors affecting human development

Variation in development has been linked to a range of variables. Geography and the associated environmental variation has often been cited as a factor that may limit or impede development [4,5,8]. For example, the problems posed by lack of waterways for inland, continental areas [8–10], and the difficulties of the tropical climate [5,8] have been cited as reasons why certain areas have had low levels of human development. Early authors infamously suggested that genetic inheritance dictates human development [11], and some continue to do so [12], but such work is generally held to be fundamentally flawed [13–15]. There is some evidence that intermediate levels of genetic diversity may positively affect development [16], though this remains highly controversial [17]. Correlations found between economic development and genetic relatedness may rather be due to cultural and geographical proximity being well predicted by genetic distance [18,19].

Cultural factors, in the form of social norms, institutions and religions have also been cited as potentially affecting economic growth and human development [20]. For example, both formal (legal) and informal (social) rules such as property rights and the structure of the institutions of government have been widely linked to development outcomes [21–23]. Others have characterized systems of governance such as centrally planned economies and dictatorships as inefficient and leading to lower economic performance [24,25], although the reasons for this are complex and multifaceted [26]. Religion has been suggested as another factor underlying relative economic performance of different countries, with Protestantism famously suggested as driving strong growth in northern Europe by Weber [27,28], and more recent studies specifically investigating this issue [29,30].

#### 1.2.1. History and human development

The impact of history on current socioeconomic development has been increasingly investigated and acknowledged: the importance of history is now little debated, and means to quantify and assess this importance are being refined [31]. Initial work in this field focused on the continuing effects of colonialism on modern post-colonial countries [32–36], but has expanded to investigate topics ranging from the effects of historic borders on government quality measured by vaccination levels [37] to the impact of the Habsburg Empire on modern European development [38]. Increasingly comprehensive datasets, especially for more limited geographical areas, have allowed robust statistical assessment of the importance of historical factors [39,40]. Observation of the intransigence of economies following a suboptimal period of extreme historical extraction has led to the formulation of the idea of multiple equilibria, where historical trauma can push an economy to a suboptimal, but stable, equilibrium, from which it is difficult to escape [41]. The effects of particular historical incidents on trajectories of socioeconomic development—path dependence—has also been documented [42].

History also interacts with geographical and cultural factors [31]. Examples include the hypothesis that geographical access to Atlantic trade routes facilitated development of more effective cultural institutions in Western Europe [43], or the continued effects on local economies of the type of administration during colonial rule in India, when geographically favourable areas were preferentially placed under direct rule [40]. Such interactions complicate the task of identifying key variables, and emphasize the importance of taking multiple factors into account.

##### 1.2.1.1. The role of shared cultural history and cultural relatedness

One aspect of history, the development impacts of which have begun to be increasingly investigated, is cultural similarity and relatedness; cultural closeness may lead to similar socioeconomic outcomes, and may facilitate intercultural spread of traits. Spolaore & Wacziarg [18,19] investigated the correlation of genetic relatedness between societies with economic indicators. They found that genetic distance from the ‘world technological frontier' (Britain in the nineteenth and the USA in the twentieth century) was negatively correlated with economic prosperity, and hypothesized that this effect was the result of easier cultural dissemination between more closely related societies.

Another approach to investigate the effect of cultural relatedness that has begun to be relatively widely employed in cultural anthropology [44–52]—but little applied to economic or developmental data—is the use of language phylogenies to capture ‘deep cultural ancestry’—i.e. the vertical pattern of descent between cultures, often spanning hundreds of years. As cultures diverge, so too do their languages, making language ancestry a useful proxy for cultural relatedness [52–54]. Recently, tools from evolutionary biology have been applied to linguistic data to infer dated family trees of the world's major language families [55–59]. This approach compares the words for basic meanings across languages and models language change as the gain and loss of homologous words (or ‘cognates') along the branches of a phylogeny, a process analogous to the gain and loss of homologous genes or morphological features on a species tree [60]. The resulting language family trees capture deep cultural connections between the cultures concerned, and have been successfully used to predict variation across socio-cultural traits in traditional [47,51,61] and modern societies [52] and to test hypotheses about patterns of cultural evolution through time [48–50].

### 1.3. Aims and approach of the current study

The aim of the current work is to investigate the application of cultural phylogenies derived from linguistic data, as has been successfully applied in cultural anthropology, to the most widely used development indicator—the HDI and its subcomponents—to assess whether deep cultural ancestry impacts major aspects of human development. To do this we use a phylogenetic generalized least-squares approach [62], initially developed in evolutionary biology, to simultaneously quantify and control for the effects of phylogeny, geography and other cofactors.

We use a modified version of Bouckaert *et al.*'s [58] phylogeny of the Indo-European languages as a proxy for cultural relatedness. While the precise timescale of Indo-European diversification remains controversial, the ancestral relationships represented in the phylogeny span a range stretching back as far as 6000–9000 BP [58]. We test for phylogenetic signal in the components of the United Nations HDI for Indo-European speaking Eurasian countries.

Alongside the impact of deep culture, we take into account three other control factors potentially linked to development, namely the role of geographical proximity, the prevalence of four major religious traditions, and whether that country had, or continues to have, a communist dictatorial government. The latter two variables have played important roles in the cultural history of Indo-European speaking countries, and have been hypothesized or shown to affect human development indicators. Including these predictors in our analysis allows us to control for the impact of more recent religious or political trends on development. Including geographical distance in our analysis allows us to control for geographical patterns that can arise due to regional ecological differences and the horizontal diffusion of ideas. In addition, we simultaneously quantify the relative importance of these successive layers of cultural influence—deep cultural ancestry, geographical proximity, religious history and a history of communism—in order to assess their relative impact in cases where more than one factor may be of importance.

## 2. Material and methods

### 2.1. Data

#### 2.1.1. Development indicators

Development data in the form of the HDI and its components were collected from the 2013 United Nations Human Development Report ([2]. The UN created the HDI as an alternative to gross national income (GNI) to measure of the level of development of a country [1]. HDI is made up of three equally weighted subcomponents: (1) an income index, a standardized transformation of GNI *per capita*; (2) a health index, a standardized transformation of life expectancy at birth and (3) an education index, a standardized combination of mean and expected years schooling. For each country in our sample, we collected data on HDI as well as its three subcomponents.

In order to minimize the effects of short-term fluctuations in development indicators, we take a country's score for each index as the average score of the years given in the 2013 Human Development Report ([2], table 2: Human Development Index trends, 1980–2012) which includes the years 1980, 1990, 2000 and 2005–2012. An analysis of variance shows country explains between 87% and 99% of the variance in indices across years (HDI, *F* = 1314.0, *η*^2^ = 0.995, *p* < 0.001; income index, *F* = 577.5, *η*^2^ = 0.986, *p* < 0.001; health index, *F* = 73.0, *η*^2^ = 0.877, *p* < 0.001; education index, *F* = 1475.9, *η*^2^ = 0.996, *p* < 0.001), justifying this approach. To correct for broad trends through time, each year's data are standardized across countries before averaging. In addition, we excluded years for which data were missing for more than one country. The resulting data matrix is recorded in electronic supplementary material, table S1.

#### 2.1.2. Language ancestry

In order to quantify and control for the effects of deep cultural ancestry, we matched countries in the UN development dataset to languages from the Indo-European language study of Bouckaert *et al*. [58]. This allowed us to use the cognate data from Bouckaert *et al*. as the basis of our phylogenies; we conducted a new analysis based on these data because we wished to add additional languages (see below) while obtaining a full posterior sample of trees. Since linguistic (and cultural) borders are less well defined than the lines that demarcate national borders, judgements had to be made about which language was most appropriate to link to each country. Our criteria for matching a country to a language were that the language was the official language or the predominant language spoken in a country. Thirty-six countries were matched to languages in this way. To maximize sample size, an additional eight Indo-European national languages not covered by the analysis of Bouckaert *et al*. were also included, because their position on the language tree is undisputed and it was relatively straightforward to incorporate them into the priors of our Bayesian phylogenetic inference procedure (see below). These eight languages were: Austrian German, Cypriot Greek, Irish English, Liechtenstein German, and Moldovan, plus the separation of Serbocroatian into Serbian, Croatian, Bosnian-Herzegovinian and Montenegrin branches. This produced a total of 44 language-to-country pairings in our analysis (electronic supplementary material, table S1).

We derive the ancestral relationships between languages based on the presence or absence of cognates (homologous words) for the languages in our sample, using the approach outlined in [58]. Cognate data for each language comprised the 200 meanings of the Swadesh word list [63]. These are basic vocabulary terms that are relatively universal and resistant to borrowing—a good example of this resistance comes from modern English, where over 50% of the lexicon consists of loanwords from Romance languages, but where this figure falls to around 6% for the Swadesh 200 word list [64].

Following previous computational approaches to the evolution of languages [55,58], we model language change as the gain and loss of cognates through time, using Bayesian phylogenetic inference as implemented in BEAST 1.7.5 [65] to infer the likely set of plausible language trees. We constructed a posterior distribution of language trees relating the 44 languages in our sample. We used a covarion model with an uncorrelated relaxed clock [66] and time depth calibrated based on known divergence times (see [58]). In addition to the 36 languages for which cognate data was available, we added eight Indo-European language–country pairings that were either not represented in the Bouckaert *et al*. [58] Indo-European phylogeny or that involved a language shared with another country. These additional languages were added to the analysis based on historical evidence of approximate divergence events with each language's closest relatives (see electronic supplementary material, table S2).

**Figure 1.**
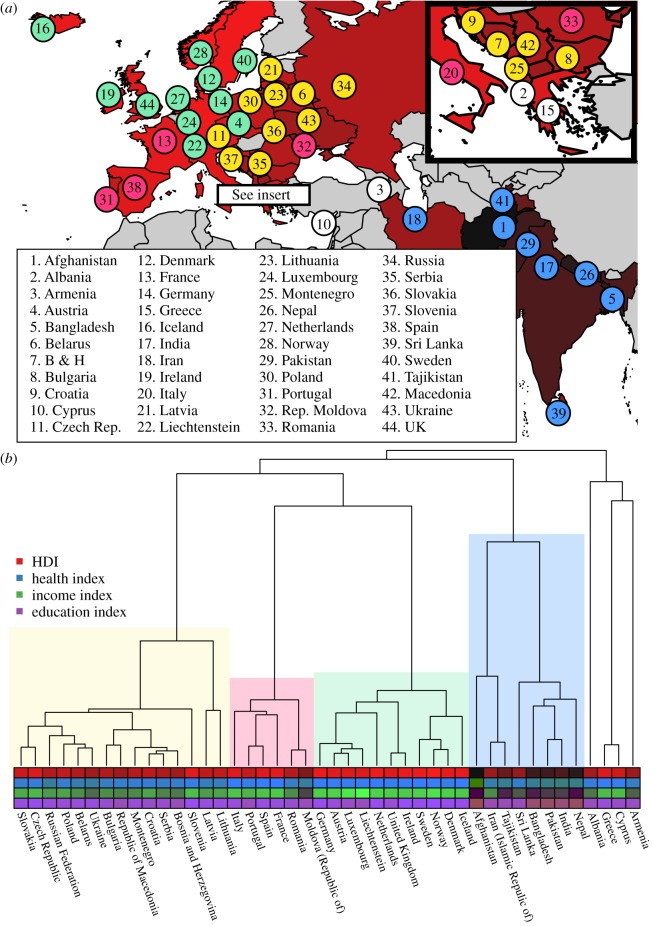
(*a*) Map showing the location of the 44 countries in our sample. Country regions are coloured according to HDI score from darker (low) to brighter (high). Coloured circles indicate major linguistic sub-groups—Germanic (green), Balto-Slavic (yellow), Italic (pink), Indo-Iranian (blue) and other (white). (*b*) Maximum clade credibility tree of 44 Indo-European languages corresponding to the countries in our sample, based on a Bayesian posterior sample of 1000 trees. The tips of the tree are colour coded according to HDI and its three subcomponents from darker (low) to brighter (high). Coloured boxes indicate major linguistic sub-groups as in panel (*a*).

We ran five separate Markov chains for 100 million iterations, sampling every 10 000th tree. The first 20 million iterations of each chain was discarded as burn-in. Examination of parameter traces in BEAST's Tracer tool indicated that the analyses had reached convergence by this point and yielded effective sample sizes of at least 100. From the total post burn-in sample of 400 million trees we randomly sampled a set of 1000 trees for analysis. Figure 1 shows the location of the countries in our sample and a maximum clade credibility tree with HDI data mapped onto the tips.

#### 2.1.3. Religion and communist history

In order to account for the effect of religious history of the countries in our sample, we incorporated the percentage of adherents to each of five religious groups—Catholicism, Protestantism, Eastern Orthodoxy, Islam and ‘Other'—as a predictor variable. The sample did not include enough countries with large enough numbers of adherents to the religions in the ‘Other' category to make subcategorization meaningful. Islam was not subdivided into, for example, Shī‘ah and Sunnī branches because no hypotheses have been proposed relating these sects to economic or developmental indicators (unlike with Protestantism—see above), and because only one country in the sample—Iran—is majority Shī‘ah.

We included an additional binary variable indicating the presence of rule of the majority of the country by a self-professed communist party, currently or in the country's history [67]. Germany was treated as non-communist because the majority of the country never experienced communist rule (only development data for the Federal Republic of Germany were used). These data are recorded in electronic supplementary material, table S1.

### 2.2. Analysis

We adapt an approach developed in evolutionary biology by Freckleton & Jetz [62] to jointly estimate the covariance of cultural ancestry and geographical proximity with development indicators, and the relative effects of our religion and communism covariates. This method uses a version of phylogenetic generalized least squares (PGLS [68]) that incorporates, alongside conventional regression covariates, information on both the phylogenetic and geographical distances between data points. This allows us to simultaneously quantify and control for the degree to which cultural ancestry and spatial proximity explain covariation in development outcomes. This approach has already been applied to cross-cultural data in the Pacific to predict cultural variation in forest outcomes across islands [51].

Two parameters, *λ* and *ϕ*, ranging between 0 and 1 represent the influence of cultural ancestry and geographical proximity effects respectively and are used to modify the standard variance matrix (***V***) according to the following formula:

V(λ,ϕ)=(1−ϕ)[(1−λ)h+λΣ]+ϕW,

where *λ* represents the magnitude of non-independence in the outcome variable due to shared ancestry effects and *ϕ* represents the same for spatial effects. **Σ** is an *n *×* n* matrix comprising the shared path lengths on the phylogeny. This is proportional to the expected variances and covariances for traits under a Brownian motion model of evolution [69]. We construct **Σ** using pairwise phylogenetic divergence from the Indo-European language phylogeny. ***W*** is the spatial matrix comprising pairwise distances between sample locations. We calculate ***W*** as the distance between the capital cities of each country based on the haversine formula [70]. ***h*** is formed from the diagonal of **Σ**, which is the distance from the start of the phylogeny to each terminal node. In our data ***h*** is constant because there are no extinct languages in our phylogeny.

Following Freckleton & Jetz [62], we can rewrite the above equation as:

$$V(\lambda ,\phi )=\gamma h+\lambda^{\prime }\Sigma +\phi W,$$

where *γ* = (1 − *ϕ*)(1 − *λ*) is the proportion of covariance independent of phylogeny and space, *λ*^′^ = (1 − *ϕ*)*λ* is the proportion of modelled covariance attributable to phylogeny and *ϕ* is the proportion of modelled covariance attributable to spatial effects. Thus covariance is a weighted sum of components proportional to shared cultural ancestry (*λ*^′^), geographical proximity (*ϕ*), and a random component (*γ*). We note that *λ*^′^ and *ϕ* should be interpreted as the proportion of modelled covariance in the outcome variables, not as a proportion of overall variance explained. *λ* and *ϕ* are determined by maximizing the log-likelihood of the phylogenetic least-squares regression model. Parameter values are estimated using an exhaustive grid search of the parameter-space which explores all values between 0 and 1 with 0.02 increments. The significance of phylogenetic and geographical influence is tested using a likelihood ratio test between models fitting both *λ* and *ϕ*, only *λ*, only *ϕ*, or neither *λ* nor *ϕ*.

Coefficients are estimated using maximum likelihood. We modelled the fixed effects of communism and the four religions as the direct effect of each of the dummy variables on each dependent variable within the PGLS framework [62]. In order to account for uncertainty in the ancestral relationships between Indo-European languages, all analyses incorporating *λ*^′^ were run across the Bayesian posterior distribution of 1000 Indo-European language trees. Where phylogeny was included in the model, we report mean and 95% confidence intervals for parameter estimates across the posterior distribution of trees.

Data were analysed in three phases. First, to explore general patterns in the data we report the bivariate effects of each predictor on each development indicator. Second, because we wished to test the effect of phylogeny against one of the most commonly cited causal factors in human development—geographical location—we fitted a single regression jointly estimating the effect of both phylogeny and geography. Third, we included communism and religion, as two important recent cultural variables, alongside phylogeny and geography in the jointly fitted regression, to simultaneously estimate the effects of all these factors. All analyses were conducted in the R v. 3.0.1 statistical package [71]. Routines for PGLS-spatial are available on request from the authors of Freckleton & Jetz [62].

Finally, cultural ancestry and geographical proximity themselves covary, because cultures expand across the landscape. The resulting collinearity may make it impossible to differentiate between covariation due to cultural ancestry and geographical proximity. In the text of the electronic supplementary material, we check whether this applies to our sample of Indo-European speaking nations, by simulating the evolution of continuous traits with varying degrees of phylogenetic signal on the Indo-European tree. This analysis shows that the approach we use here can reliably identify traits that show covariation with geographical proximity or cultural distances. We also show that the method correctly identifies an archetypal geographically autocorrelated trait (mean temperature) as related to geographical proximity and not phylogeny (see electronic supplementary material).

## 3. Results

Table 1 reports bivariate statistical tests for a relationship between the development indicators and deep cultural ancestry (*λ*) or geographical proximity (*ϕ*), as well as religion and communism. We find strong phylogenetic signal in all of the development indicators (*λ* > 0.89 across indicators)—i.e. across the phylogeny, development scores covary strongly with cultural ancestry. Figure 1 illustrates this relationship, showing HDI and its subcomponents mapped onto the Indo-European language phylogeny. Table 1 also reveals a strong geographical signal (*ϕ* > 0.97 across indicators), positive effects of Protestantism and Catholicism and negative effects of Islam across all indicators, as well as negative effects of communism on the income and health indices. Pairwise correlations between all predictors are shown in electronic supplementary material, table S3.

**Table 1.** Results of bivariate regression predicting HDI and its subcomponents. The first two rows show mean estimates from an intercept-only PGLS analysis testing for either cultural ancestry (*λ*) or geographical distance (*ϕ*) effects. We used likelihood ratio tests to evaluate support for these models against a null model with no ancestry or geographical proximity effects. The remaining columns show mean, 95% confidence intervals and *p*-values of the β-coefficients from ordinary least-squares (OLS) bivariate regressions of each predictor on each development indicator. Significance levels: * < 0.05; ** < 0.01; *** < 0.001.

predictor		HDI	income index	health index	education index
shared ancestry (*λ*)	mean	0.975***	0.895***	0.999***	0.944***
	95% CI	(0.823, 1.00)	(0.637, 1.00)	(0.930, 1.00)	(0.741, 1.00)
geographical proximity (*ϕ*)	mean	0.980***	0.980***	0.980***	0.960***
	95% CI	(0.935, 1.00)	(0.950, 1.00)	(0.865, 1.00)	(0.870, 1.00)
*Protestant*	mean	0.0157**	0.0154**	0.0133*	0.00274***
	95% CI	(0.0136, 0.0179)	(0.0132, 0.0176)	(0.0111, 0.0155)	(0.00235, 0.00313)
*Catholic*	mean	0.0127***	0.0146**	0.0120***	0.00161*
	95% CI	(0.0112, 0.0142)	(0.0132, 0.0161)	(0.0105, 0.0135)	(0.00132, 0.0019)
*Orthodox*	mean	−0.0011	−0.00359	−0.00216	0.000462
	95% CI	(−0.0029, 0.0007)	(−0.00538, −0.0018)	(−0.00393, −0.000384)	(0.00014, 0.000784)
*Muslim*	mean	−0.0210***	−0.0192***	−0.0189***	−0.00383*
	95% CI	(−0.0225, −0.0195)	(−0.0208, −0.0176)	(−0.0205, −0.0174)	(−0.0041, −0.00356)
*communism*	mean	−0.397	−0.653*	−0.701*	0.0144
	95% CI	(−0.515, −0.279)	(−0.767, −0.539)	(−0.811, −0.590)	(−0.00714, 0.0360)

Table 2 shows phylogeny and geography analysed together in a multivariate PGLS [62] framework, where both variables are fitted simultaneously, thereby controlling for one another. Table 2 indicates that geography is a significant predictor of HDI and all its subcomponents, whereas phylogeny is a minor but significant predictor of health and income indices.

**Table 2.** Results from multivariate PGLS regressions predicting HDI and its subcomponents, incorporating phylogenetic and spatial effects. The table shows mean and 95% highest posterior density (HPD) of shared ancestry (*λ*′) and geographical distance (*ϕ*) effects across the posterior sample of 1000 trees. Significance tests reflect the mean *p*-value across the posterior distribution of trees and incorporate both stochastic and phylogenetic uncertainty. Significance levels: * < 0.05; ** < 0.01; *** < 0.001. .

predictor		HDI	income index	health index	education index
shared ancestry (*λ*′)	mean	0.0482	0.0352**	0.2850***	0.0009
	95% HPD	(0.005, 0.118)	(0.02, 0.04)	(0.22, 0.34)	(0.00, 0.00)
geographical proximity (*ϕ*)	mean	0.943***	0.965***	0.715**	0.959*
	95% HPD	(0.88, 0.98)	(0.96, 0.98)	(0.64, 0.76)	(0.96, 0.96)

Table 3 shows results when all predictors—including religion and communism—are analysed simultaneously using the PGLS framework. This reveals that deep cultural ancestry does not significantly predict any response variable after controlling for other factors. Geographical proximity significantly predicts similarity in HDI and income index. Communism was significantly negatively associated with income index, health index and HDI as a whole, but not education index. There was no significant effect of Protestantism, Catholicism or Orthodox Christianity but Muslim countries showed significantly lower scores on education index.

**Table 3.** Results from multivariate PGLS regressions predicting HDI and its subcomponents, incorporating phylogenetic and spatial effects and religion and communism independent variables. The table shows median and 95% HPD of shared ancestry (*λ*′) and geographical distance (*ϕ*) effects across the posterior sample of 1000 trees. Also shown are *β* estimates and 95% HPD for the religion and communism predictors, averaged across the posterior sample of 1000 trees. Significance tests reflect the mean *p*-value across the posterior distribution of trees and incorporate both stochastic and phylogenetic uncertainty. Significance levels: * < 0.05; ** < 0.01; *** < 0.001.

predictor		HDI	income index	health index	education index
shared ancestry (*λ*′)	mean	0.0055	0.0104	0.513	0.0862
	95% HPD	(0.00, 0.00)	(0.00, 0.0128)	(0.00, 1.00)	(0.00, 0.90)
geographical proximity (*ϕ*)	mean	0.975**	0.982***	0.486	0.851
	95% HPD	(0.98, 0.98)	(0.98, 1.00)	(0.00, 0.96)	(0.00, 0.94)
*Protestant*	mean	0.00092	0.00183	0.00307	0.00081
	95% HPD	(−0.00010, 0.00284)	(0.00014, 0.00351)	(0.00071, 0.00543)	(0.00037, 0.00125)
*Catholic*	mean	−0.00026	0.00234	0.00075	−0.00005
	95% HPD	(−0.00156, 0.00105)	(0.00126, 0.00341)	(−0.00049, 0.00198)	(−0.00037, 0.00027)
*Orthodox*	mean	−0.00306	−0.00127	−0.00419	−0.00017
	95% HPD	(−0.00440, −0.00172)	(−0.00244, −0.000101)	(−0.00561, −0.00276)	(−0.00048, 0.00015)
*Muslim*	mean	−0.00584	−0.00339	−0.00270	−0.00145*
	95% HPD	(−0.00699, −0.00468)	(−0.00444, −0.00234)	(−0.00403, −0.00137)	(−0.00172, −0.00118)
*communism*	mean	−0.518**	−0.792*	−0.768***	0.00055
	95% HPD	(−0.587, −0.449)	(−0.860, −0.725)	(−0.890, −0.645)	(−0.0165, 0.0176)

Since communism predicted both income index and health index (table 3), which are themselves correlated (electronic supplementary material, table S3), we ran a follow-up analysis including income index as an additional predictor of health index, in order to determine whether national income mediates the effect of communism on health. This indicates that communism is no longer a significant predictor of health index (*β*_communism_ = −0.25, *p* = 0.478) when controlling for income index (*β*_income index_ = 0.507, *p* < 0.01).

## 4. Discussion

### 4.1. Cultural ancestry alone predicts human development

The bivariate analyses presented in table 1 indicate that, in the absence of any other control variables, deep cultural ancestry strongly covaries with HDI and its components, income index, health index and education index. Language phylogenies alone are thus able to account for a substantial portion of variation in development indicators between countries. While this could arise due to direct inheritance of the traits in question, in the case of HDI it appears more likely to be due to the presence of omitted predictor variables that are themselves correlated with the phylogeny due to cultural inheritance, biased diffusion or chance. An analogy can be made here with biology, where species phylogenies are powerful predictors of variation in many traits, including those not inherited in the conventional sense [72]. For example, latitude and rainfall covary with species phylogenies due to a combination of habitat selection and the fact that one generation tends to be born into an environment similar to the previous generation [72]. As a result, regardless of the mechanism of inheritance, species phylogenies predict unmodelled variation in a range of traits across taxa [68]; our findings show that the same may be true of cultural phylogenies.

### 4.2. Incorporating geography

Geography alone is also a significant predictor of HDI and its components (table 1). When included in the model alongside cultural phylogeny, geography remains a significant predictor for all response variables (all *ϕ* > 0.7), while phylogeny remains a significant predictor of income and health indices, although its effect is small (*ϕ* of 0.28 and 0.04, respectively). This indicates geography is more important than cultural ancestry for all aspects of the HDI and accords with the great importance of geography previously highlighted by many authors [4,5,8–10] (see §4.2.1 below).

#### 4.2.1. The effect of geography on Human Development Index

The influence of geography on HDI found potentially corroborates a large body of work documenting effects of climate, natural resources, and transportation and communication barriers on economic growth and development [5,9,10,73,74]. The Indo-European languages span a large geographical area with climates ranging from boreal to tropical [75]. Tropical diseases can negatively impact longevity [5] and may help explain the poorer development indicators of tropical and subtropical countries, represented in our dataset by India, Bangladesh, Sri Lanka and Pakistan (although HDI is relatively high in Sri Lanka). Weather-related disasters are also more prevalent in tropical areas, with tropical cyclones causing more deaths than any other natural hazard [76] alongside infrastructure damage which further impedes development [77]. Large arid inland areas in Afghanistan, Pakistan, Tajikistan and Iran (see [75]) may also impede development, as lack of sea access can limit trade [78], and aridity can impede food and water supply [79].

It should also be noted, however, that horizontally transferred (i.e. borrowed from other cultures, rather than inherited) cultural traits may also contribute to the importance of geographical proximity. Cultural traits should be more easily and frequently transferred between neighbouring countries. Examples of the spread of such traits are abundant historically, with, for example, industrialization spreading rapidly into Western Europe from Great Britain [80], but taking much longer to begin in Russia [81]. Even if improved transport and communications may make policymakers increasingly aware of policies in countries far away from their own, the spread of cultural traits between populations as a whole should be more likely between neighbouring countries. Distinguishing between the effects of environment and communication distance requires further work (the focus here was not geography), although the distance matrix used is a better approximation of physical distance than of climatic dissimilarity between countries.

#### 4.2.2. Phylogeny continues to predict health and income index

Controlling for geographical proximity, phylogeny continues to be a minor, but significant predictor of health and education indices. Such a pattern could indicate vertical inheritance of traits linked to these development outcomes, or may be due to phylogeny covarying with other unaccounted- for variables. Possible candidates for such variables are borrowed cultural influences (e.g. political system, or religion—see §4.3), environmental factors or genetics. Cultural factors are perhaps more likely, because genetic proximity in Europe, and environment, are already well predicted by geography [82]. There remains, however, the possibility that a small part of genetic proximity varies with the phylogeny, and that this affects, for example, longevity; further work would be required to completely exclude this possibility, and would be of interest given that genetics are likely to influence longevity in particular [83,84]. Furthermore, Spolaore & Wacziarg [18,19] indeed find a correspondence between genetic relatedness and economic indicators, but attribute this to cultural inheritance—disentangling both factors fully is an avenue of future research.

If cultural phylogeny were to really exert an influence on health and income, direct inheritance of the traits involved is unlikely because most innovations underlying modern income and health metrics are far more recent than most splits in the language phylogeny. Two alternative explanations are (i) underlying inherited cultural traits facilitated parallel development of structures increasing both metrics (e.g. industrial production, powered transportation, sanitation) in more closely related societies, or (ii) borrowing between more closely related cultures faces fewer impediments, and thus innovations spread more rapidly into more closely related than more distantly related cultures. There is evidence for both possibilities [85], but in the European context, borrowing would have been likely to have been more important as key innovations such as industrialization [86], mass education [87] and social insurance [88] are documented as originating in one country and spreading, not evolving independently. This would accord with recent work by Spolaore & Wacziarg [18,19] positing that cultural distance from the ‘world technological frontier' and barriers to adoption of new innovations explain the effects of genetic distance on economic development and other work indicating that cultural and linguistic relatedness predicts aspects of the economic performance of nation states [52].

### 4.3. Incorporating recent cultural factors—religion and communism

Our bivariate analyses show that the recent cultural factors examined—communist history and religion—are, taken alone, good predictors of the Human Development Index and its components (table 1). When incorporated alongside phylogeny and geography, phylogeny ceases to be a significant predictor of HDI or any of its components, meaning recent cultural factors combined with geography can account for covariation between HDI and cultural phylogeny (table 3). Communism significantly negatively predicts HDI, income and health indices, but religion ceases to be a significant predictor except for a negative correlation between Islam and education index. These results support a significant effect of communist history on the human development of countries, comparable to the effects of geography (which remains a significant predictor of HDI and income index), and more immediately important than cultural phylogeny or religion.

#### 4.3.1. Communism and religion as mediators of cultural ancestry

Together with geography, recent cultural factors—especially communist history—provide a better explanation than deep cultural phylogeny for HDI values. However, it is worth noting that these recent cultural factors may in part mediate the relationship between HDI and cultural phylogeny—i.e. rather than communism and religion explaining away the effects of deep cultural ancestry on modern cultural variation, they may in fact be an example of it.

Electronic supplementary material, table S3 shows that communism itself covaries strongly with deep cultural ancestry. All Balto-Slavic-speaking countries in the dataset were at one point communist. This is in large part due to the political influence of Russia—both the Russian Empire [89] and subsequently the USSR [90]—on the surrounding countries, which, unsurprisingly, often spoke related languages. However, it is probable that cultural and linguistic similarity played a role in the continued influence of Russia on Slavic-speaking countries in the Balkans and Eastern Europe. For example, after World War II, Greece entered the Western bloc, while neighbouring Slavic-speaking Bulgaria and Yugoslavia entered the Eastern bloc and were previously long allied to Russia [90,91]. Similarly, Slavic-speaking Czechoslovakia entered the Eastern bloc whereas neighbouring largely Germanic-speaking Austria entered the Western bloc [90]. While these events were, of course, due to political decisions and conflict outcomes, they can still be looked upon as part of the documented pattern of generally stronger cultural ties between linguistically similar countries [52].

Similarly, three of the four religion variables covary with deep cultural ancestry (electronic supplementary material, table S1). All countries with Protestant majorities, and most with large (greater than 10%) Protestant populations, in the dataset are Germanic-speaking, and while this correspondence is not clear-cut (e.g. Slovakia and Latvia have a large Protestant population), the pattern of spread/retention of religions is plausibly attributable to linguistic and other aspects of deep cultural similarity facilitating their spread and maintenance. While social change is not strongly impeded by deep cultural phylogeny, exchange of ideas may well be facilitated by it.

#### 4.3.2. The effect of communism on Human Development Index

Communist history shows a significant negative correlation with the national income of the countries in our dataset. Post World War II economic growth in communist countries was modest, especially during the 1970s and 1980s, relative to non-communist European countries [92], and the centrally planned economy of communist countries has long been held by economically liberal theoreticians to hamper conventional economic growth [93–95]. Although the countries in the dataset had abandoned communism for most of the years in the dataset, the residual effect of communism appears to still be detectable. Institutional and cultural traits produced by communism and by dictatorship may continue to retard growth today, with corruption still regarded as higher in Eastern than Western Europe [96] and linked to lower national income [97]. It should also be noted, however, that many of the former communist countries (largely those in the former Soviet Union) also suffered major economic turmoil following the demise of their communist governments [98], and that this too may play a role in explaining the apparent effect of communism on income. Moreover, it must be noted that the communist countries in the sample are all Eastern European and Central Asian, and that these areas were less wealthy than Western Europe even prior to communism [92,99], and indeed Russia saw rapid economic growth following the advent of communism, although this lessened over time [92,100]. For all these reasons the results presented here must be treated with caution, and are primarily intended as a control in the context of examination of deep cultural effects on human development, not as a thoroughgoing analysis of the effects of communism on development.

Communism also shows a significant negative association with health index (i.e. normalized longevity), although only at *p* = 0.05 level. This confirms the stagnation and even decline of life expectancy in Europe under communism during the 1970s and 1980s, corresponding to years of low economic growth (see above), which has continued to set formerly communist countries back in terms of life expectancy until today [101,102]. The proximate causes for this low life expectancy are complex, but high alcohol consumption, smoking and poor workplace safety, as well as low quality diet and living conditions associated with lower income levels are implicated [101]. Most of the same caveats also apply here as to the economic effects of communism however, with lifespan decreasing rapidly in the former Soviet Union immediately following post-Soviet collapse [101], and lifespan having increased strongly in the Soviet Union prior to and immediately after World War II [103].

Longevity greatly increased during recent centuries in Europe in part due to generally rising living standards (and thereby nutrition [104]), with increasing health and longevity interacting with the economy in a positive feedback loop [105]. Communist history may thus have also influenced longevity via its effect on income, with income being a significant predictor of health index (electronic supplementary material, table S1). Consistent with this explanation, we find that communism is no longer a significant predictor of health index when controlling for income index.

#### 4.3.3. The effect of religion on Human Development Index

The only correlation between religion and human development which remains in the model including all predictor variables is a negative of Islam with education index. This may be due to lower female enrolment—with education largely restricted to males in many Islamic societies at the start of the twentieth century [106]—or due to a traditional focus on religious rather than secular education [107]. Alternatively, the correlation may be spurious. The nature of the dataset means that three majority Muslim countries have very low education indices (Afghanistan 0.306, Bangladesh 0.377, Pakistan 0.353, sample average 0.719), and Iran also shows a below-average education index (0.618). However, education level is also low in neighbouring India (0.433) and Nepal (0.348), which are predominantly Hindu (81.3% and 79.8% respectively, although 14.2% of India's population are Muslim [67]), but which share many non-religious aspects of cultural history.

The female : male secondary education ratio is lower than most of the sample for the South Asian Muslim countries and Iran, and may be connected to patriarchal social structures [108]. Whether this can be directly attributed to Islam *per se* is questionable, however. In the sample, several countries with majority or very large (greater than 30%) Muslim populations have very high education indices and female enrolment (Albania, Macedonia, Tajikistan), although this may partly be due to a mitigating effect of communist history (which is factored out in our joint analysis), with relatively high educational level given the income level in many Eastern bloc countries [109], especially of women [110], and a positive (although non-significant) association found between communism and education in this sample. However, globally, several predominantly Muslim countries with no communist history today have education levels and female enrolment at least as high as similarly developed predominantly non-Muslim countries; the current education index of Brunei and Saudi Arabia is higher than that of Portugal and close to that of Spain and Greece, while the education index of women in Qatar, the United Arab Emirates and Brunei is higher than that for men, and the female : male education index ratio of the latter three countries is higher than in Spain, Portugal or Greece (see electronic supplementary material, table S4). A wider-scale and more nuanced analysis may allow firmer conclusions.

Interestingly, after controlling for other covariates, we find no effect of Protestantism on development, *contra* the famous hypotheses of Weber [27,28] and some more recent empirical work [29,111]. Protestantism was historically specifically contrasted with Catholicism, which was hypothesized to impede economic growth. However, in our dataset some predominantly Catholic countries were among the best performing economically (Luxembourg and Austria were the second and fifth highest income indices, respectively, ahead of six and four predominantly Protestant countries, respectively), and the data examined here provides no solid evidence supporting the idea of Protestantism improving economic welfare. It should perhaps, however, be noted that several countries with a historically very strong Protestant tradition (Czech Republic, Germany, The Netherlands, United Kingdom) now have large (greater than 20%) non-religious populations (see electronic supplementary material, table S1), which may mask a historic economic impact of Protestantism.

### 4.4. Conclusion

Our analysis is the first to combine high-resolution language phylogenies with PGLS to simultaneously examine the effects of cultural phylogeny, geography and recent cultural factors on development. Our results show that while deep cultural ancestry alone can predict variation in HDI indicators, this relationship is better attributable to the effect of geography and recent cultural changes, which emerge as more important predictors of human development. The absence of a strong independent effect of cultural ancestry is a broadly hopeful conclusion for policymakers, for it indicates that development outcomes are not tightly constrained by deep cultural inheritance and are, therefore, more likely to be amenable to policy change. However, our findings also add to the large body of literature underlining the importance of a potentially less malleable factor—geography—in affecting development. Development outcomes are likely to be constrained by geography to the extent that the geographical proximity effects we identify reflect direct environmental influence. The effect of geography may, however, be due to its influence on cultural diffusion, and, therefore, geographical impediments may be increasingly overcome by improved communication. Nevertheless, the potential effect of deep cultural ancestry should not be entirely ignored and should continue to be tested; cultural phylogeny is closely associated with HDI values, and although recent cultural developments have a more important role than deep cultural phylogeny in development outcomes, as we note above, the spread and maintenance of these developments themselves may be facilitated by linguistic and cultural phylogenetic proximity. It should also be noted that the focus on a relatively small sample of 44 countries speaking Indo-European languages may have limited our statistical power to detect effects that are in fact present—potentially including a cultural phylogenetic effect—particularly in the multivariate analysis. Larger-scale analysis of the causal relationships between development outcomes, geography, religion, political ideology and deep cultural phylogeny around the globe would thus be a fruitful avenue of further research.

## Supplementary Material

Supplementary Text

## Supplementary Material

Supplementary Table S1

## Supplementary Material

Supplementary Table S2

## Supplementary Material

Supplementary Table S3

## Supplementary Material

Supplementary Table S4

## Data accessibility

All data generated or analysed during this study are included in this published article and its Supplementary Information files. Cognate presence/absence data used to build the language tree is available in the supplementary material of Bouckaert *et al.* [58].
