# Supplementary material for: Deep cultural ancestry and human development indicators across nation states
Source: R Soc Open Sci. 2018 Apr 11;5(4):171411. doi: 10.1098/rsos.171411 (PMC5936893; doi:10.1098/rsos.171411)
Supplement: Supplementary Text [file rsos171411supp1.pdf]

Supplementary Text

Deep cultural ancestry and human development indicators across nation states

Roland B. Sookias[1] *, Samuel Passmore[2,3], Quentin D. Atkinson[2,4] *

Affiliations:

1. Museum für Naturkunde, Leibniz Institute for Evolution and Biodiversity Science, Invalidenstraße 43, 10115 Berlin, Germany

2. School of Psychology, University of Auckland, Auckland, Private Bag 92019, Auckland 1142, New Zealand

3. Department of Anthropology and Archaeology, University of Bristol, 43 Woodland Road, Bristol BS8 1UU, United Kingdom

4. Max Planck Institute for the Science of Human History, Jena, Germany

***emails: q.atkinson@auckland.ac.nz; sookias.r.b@gmail.com**

Differentiating cultural ancestry from geographic proximity

The Phylogenetic Generalized Least Squares (PGLS) approach developed by [1] provides a method to jointly estimate the covariance of cultural ancestry and geographic proximity with a trait of interest, whilst simultaneously controlling for and estimating other fixed effects as covariates. This approach has already been applied to cross-cultural data in the Pacific to predict cultural variation in forest outcomes across islands [2]. One potential caveat when fitting models such as this is that cultural ancestry (as measured by linguistic distance) and geographic proximity are often correlated. This collinearity can make it impossible to differentiate between covariation in a trait due to cultural ancestry versus geographic proximity. The extent to which this is a problem depends on the nature of the association between linguistic and geographic distance matrices and will vary across language groups. To check whether we can reliably identify covariation with language ancestry versus geographic proximity in our data, we simulated the evolution of traits along the branches of the Indo-European language tree with varying degrees of phylogenetic signal. We then evaluated whether and to what extent the PGLS spatial method could recover the phylogenetic signal in the data and the extent to which phylogenetic signal was confounded with geographic signal. We also used an archetypal geographically autocorrelated trait (mean temperature across the continent) to test whether covariation with geographic proximity can be correctly identified and distinguished from cultural ancestry effects.

We simulated trait evolution along the brances of the Maximum Clade Credibility tree of Indo-European languages derived from the posterior sample of trees used in the main text. Traits were simulated under the assumption of Brownian motion using the `rTraitCont` function in the *APE* package [3], in *R v.3.0.1* [4], with standard deviation set to 0.1. In order to evaluate the performance of PGLS spatial across varying strengths of phylogenetic signal in the data, we transformed the branch lengths of the tree using Pagel's lambda (λ) [5] transformation of branch

lengths. λ can range between 0 and 1. A λ value of 1 results in no change to branch lengths, such that trait evolution reflects a Brownian diffusion process along the branches of the tree. Smaller values of λ result in longer periods of independent evolution along the terminal branches of the tree, corresponding to weaker phylogenetic signal in the data. A transformation of 0 results in a "star-like" phylogeny in which all of the branch length is in the terminal branches, equivalent to no phylogenetic signal in the data [6,7]. We simulated data on the Indo-European language tree for λ transforms ranging between 0 and 1 with intervals of 0.2. Each simulation was run 1000 times. This produced six sets of traits comprising 1000 simulated datasets ranging from traits show no covariation with phylogeny (λ = 0) to clear covariation with phylogeny (λ = 1). In addition to data simulated on the Indo-European phylogeny, we include estimates of mean annual temperature at the location assigned to each language [8]. We take this as an archaetypal trait showing covariation with geographic proximity that could not be influenced by language ancestry. Future work could explore different types of real or simulated geographically autocorellated data, however such an analysis is beyond the scope of this paper.

Figure A shows the results of running PGLS spatial analysis on the simulated and temperature data, simultaneously inferring the relative covariance with cultural ancestry (λ) and geographic proximity (ϕ; see Methods section, main text). Table A provides the median inferred ϕ and λ values across the same six sets of simulated data, together with the percentage of simulations yielding significant values of ϕ and λ. These results show that when the true value of λ is set to 0, we correctly infer no covariance with cultural ancestry or geographic proximity. As the true value of λ in the simulated data increases, we infer proportionately greater covariance with cultural ancestry. As expected, there is a range of inferred λ values across the 1000 simulations for each value of λ. This includes the true value of λ in each case and median estimates tend to be somewhat conservative – i.e., if anything, we underestimate phylogenetic signal in the data as measured by λ. As λ approaches 1, our power to detect a significant phylogenetic effect increases to 99.8%. By contrast, estimates of covariance with geographic proximity remain at or close to zero across the range of λ

values. In the case of mean temperature, we corrrectly infer the inverse pattern, finding strong covariation with geographic proximity (ϕ = 1.00, p<0.001), and no covariation with cultural ancestry (λ = 0.00, p=1.00).

We have not considered more complex simulation models such as directional evolution, or traits that covary with both geography and phylogeny. Nevertheless, our simulations show that, at least for the sample of Indo-European cultures considered here, the association between cultural ancestry and geographic proximity does not prevent us from differentiating between variables showing clear covariance with cultural ancestry or geographic proximity. When trait data is simulated under a model of Brownian motion along the branches of the Indo-European phylogeny either untransformed (λ = 1) or transformed to reflect moderate departures from brownian diffusion (λ = 0.8 or 0.6), we can reliably recover evidence of phylogenetic signal and correctly infer low or no geographic signal. Conversely, for an archetypal geographically distributed trait (mean annual temperature), we correctly infer strong covariation due to geographic proximity and no phylogenetic signal.

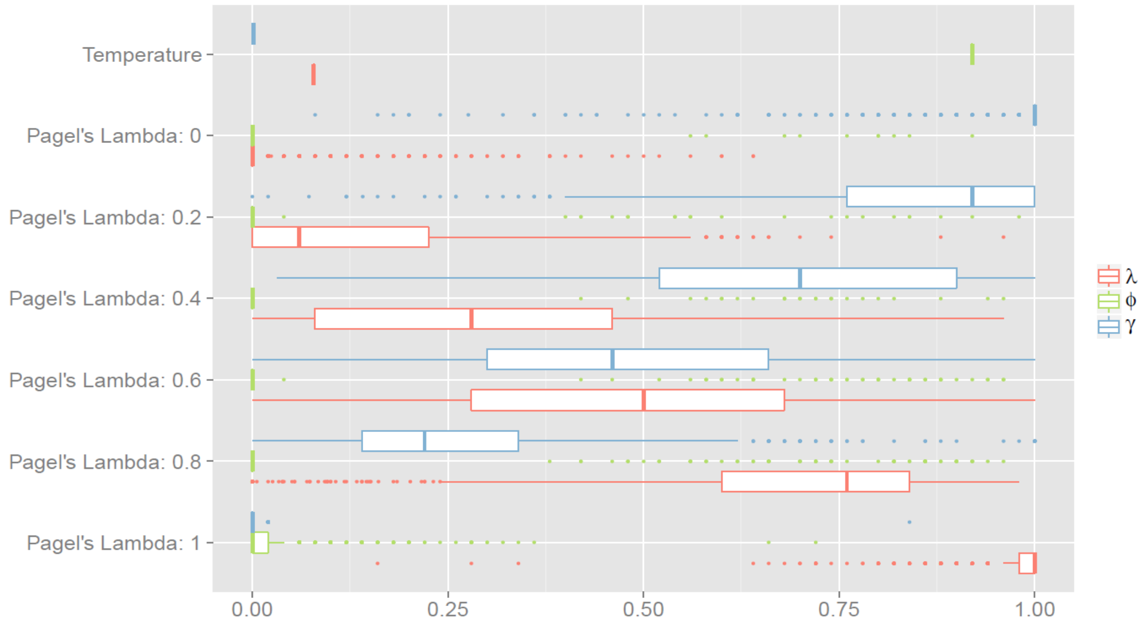

Figure A: Boxplots of PGLS-Spatial model showing inferred estimates of variance attrributable to cultural ancestry (lambda, λ, red), geographic proximity (phi, ϕ, green) and variance independent of cultural ancestry and geographic proximity (γ, blue) across the six sets of 1000 simulated datasets, plus mean temperature. Temperature results relate to one dataset and so yield a single point estimate for each parameter.

Table A: For each phylogenetically simulated variable, the mean phi and lambda score along with the percentage significant. The temperature variable shows the mean phi and lambda score and p-values for each coefficient, since there was only one iteration.

**True λ**	**Median λ'**	**% sig.**	**Median ϕ**	**% sig.**
0.0	0.00	0.6%	0.00	0.0%
0.2	0.06	14.4%	0.00	0.1%
0.4	0.28	39.0%	0.00	0.1%
0.6	0.52	65.9%	0.00	0.5%
0.8	0.76	88.0%	0.00	1.1%
1.0	1.00	99.8%	0.00	0.2%

References

1. Freckleton R, Jetz W (2009) Space versus phylogeny: disentangling phylogenetic and spatial signals in comparative data. Proceedings of the Royal Society B-Biological Sciences 276: 21-30.
2. Atkinson QD, Coomber T, Passmore S, Greenhill SJ, Kushnick G (2016) Cultural and Environmental Predictors of Pre-European Deforestation on Pacific Islands. PLoS ONE 11: e0156340.
3. Paradis E, Claude J, Strimmer K (2004) APE: analyses of phylogenetics and evolution in R language. Bioinformatics 20: 289-290.
4. Team RC (2014) R: A language and environment for statistical computing. Vienna, Austria: R Foundation for Statistical Computing.
5. Pagel M (1999) Inferring the historical patterns of biological evolution. Nature 401: 877-884.
6. Pagel M (1999) Inferring the historical patterns of biological evolution. Nature 401: 877-884.
7. Paradis E, Claude J, Strimmer K (2004) APE: analyses of phylogenetics and evolution in R language. Bioinformatics 20: 289-290.
8. Mitchell TD, Carter TR, Jones PD, Hulme M, New M (2004) A comprehensive set of high-resolution grids of monthly climate for Europe and the globe: the observed record (1901–2000) and 16 scenarios (2001–2100). Tyndall centre for climate change research working paper. pp. 25. Available here: http://data.worldbank.org/data-catalog/cckp_historical_data
