# Supplementary material for: Deep cultural ancestry and human development indicators across nation states
Source: R Soc Open Sci. 2018 Apr 11;5(4):171411. doi: 10.1098/rsos.171411 (PMC5936893; doi:10.1098/rsos.171411)
Supplement: Supplementary Table S4 [file rsos171411supp5.doc]

**Supplementary Table S4** – Education indices of four developed, predominantly Muslim countries outside the sample used in this study compared to those of three predominantly non-Muslim countries included in the sample. Human development and education index data are for 2015 and sourced from the United Nations Human Development Report 2016, and % Muslim data are from the CIA World Factbook obtained on 8/8/2017.

|  |  |  | **Education index** | | |  |
| --- | --- | --- | --- | --- | --- | --- |
| **Country** | **% Muslim** | **Human Development**  **Index** | **Both sexes** | **Female** | **Male** | **Female:male education index** |
| ***Portugal*** | < 0.6 | 0.843 | 0.758 | 0.755 | 0.758 | 0.996 |
| ***Spain*** | < 2.2 | 0.884 | 0.818 | 0.820 | 0.817 | 1.004 |
| ***Greece*** | 1.3 | 0.866 | 0.828 | 0.818 | 0.841 | 0.974 |
| ***Qatar*** | 67.7 | 0.856 | 0.699 | 0.738 | 0.686 | 1.076 |
| ***Brunei Darussalam*** | 78.8 | 0.865 | 0.797 | 0.728 | 0.709 | 1.027 |
| ***Saudi Arabia*** | 100.0* | 0.847 | 0.767 | 0.725 | 0.806 | 0.900 |
| ***United Arab Emirates*** | 76.0 | 0.840 | 0.686 | 0.739 | 0.648 | 1.141 |

*Official estimate is 100% Muslim, but some 30% of the population is foreign, and many of this group are non-Muslim.
